# Peri-operative chemotherapy for the treatment of resectable liver metastases from colorectal cancer: A systematic review and meta-analysis of randomized trials

**DOI:** 10.1186/1471-2407-10-309

**Published:** 2010-06-21

**Authors:** Martina Wieser, Stefan Sauerland, Dirk Arnold, Wolff Schmiegel, Anke Reinacher-Schick

**Affiliations:** 1 Department of Internal Medicine, Knappschaftskrankenhaus Bochum Langendreer, University of Bochum, Bochum, Germany; 2 Institute for Clinical-Oncological Studies within P.U.R.E., University Bochum, Bochum, Germany; 3 Institute for Research in Operative Medicine, University of Witten/Herdecke, Cologne, Germany; 4 Department of Hematology and Oncology, University of Halle, Germany

## Abstract

**Background:**

The role of peri-operative chemotherapy in patients with resected stage IV colorectal cancer (CRC) remains to be defined. This study was aimed at evaluating the effectiveness of peri-operative chemotherapy in patients with resected stage IV CRC by performing a meta-analysis of relevant trials.

**Methods:**

We performed a literature search to identify trials comparing patients with stage IV CRC receiving peri-operative chemotherapy and surgery with patients undergoing surgery alone. The hazard ratio (HR) was estimated to assess any survival advantage of peri-operative chemotherapy.

**Results:**

Eight trials conducted on a total of 1174 patients were identified by a literature search. In these trials, HR estimates suggested that peri-operative chemotherapy yielded no survival advantage over surgery alone (HR, 0.94; 95%CI, 0.8-1.10; *p *= 0.43). In a subset analysis on intra-arterial chemotherapy alone, no survival benefit was evident (HR, 1.0; 95% CI, 0.84-1.21; *p *= 0.96; I^2 ^= 30%), whereas in the trials involving systemic chemotherapy, the difference between the groups approached statistical significance (HR, 0.74; 95% CI, 0.53-1.04; *p *= 0.08; I^2 ^= 0%). Both peri-operative treatment groups had a significant recurrence-free survival benefit (HR, 0.78; 95% CI, 0.65-0.95; *P *= 0.01 for hepatic arterial infusion; and HR, 0.75; 95% CI, 0.62-0.91; *p *= 0.003 for systemic therapy). The toxicities of chemotherapy were acceptable in most trials.

**Conclusions:**

This is the first meta-analysis demonstrating the importance of peri-operative chemotherapy in the treatment of resected stage IV CRC. Although the results must be carefully interpreted because of some limitations, critical issues were identified that must be resolved by future studies.

## Background

Colorectal cancer (CRC) is a leading cause of cancer-related mortality worldwide with approximately 500.000 deaths annually [1-3]. Nearly 25% of patients with CRC present with synchronous liver metastases at the time of initial diagnosis [4]. Recurrences after resection of the primary tumor will occur in 60%-70% in the liver [5].

Surgery of colorectal liver metastases remains the best treatment modality for potential cure with a 5-year overall survival (OS) rate between 25% and 40% [6-8], but at present only 20%-35% of all patients are suitable candidates for surgery [5].

The long-term survival rate, even after surgical resection, is unacceptably low, thus to improve survival of patients with resected stage IV CRC, the development of effective peri-operative therapy is essential.

Prospective randomized trials investigating the role of post-operative adjuvant chemotherapy in patients with resected CRC have been performed since the 1980 s.

Local hepatic arterial infusion (HAI) has been investigated as additive or adjuvant therapy after resection of liver metastases in patients with CRC in an effort to reduce hepatic recurrence with conflicting results regarding survival benefit [9-13].

A pooled analysis of two adjuvant systemic chemotherapy trials reported in 2008 revealed a hazard ratio (HR) for OS of 0.76 for patients treated with 5 FU/FA. The result for recurrence-free survival (RFS) was only of marginal significance and may have been due, in part, to the fact that both trials used an outdated chemotherapy regimen and had to be closed prematurely because of slow accrual [14]. Subsequently, one randomized trial investigating the role of peri-operative chemotherapy using a more active chemotherapy regimen and a larger number of accrued patients has been conducted [15].

This study failed to demonstrate a significant improvement in recurrence-free survival for the peri-operative chemotherapy group in the intent-to-treat population.

Therefore, we performed a meta-analysis using data from all these trials to determine the effect of peri-operative chemotherapy on overall survival and recurrence-free survival in patients with resected stage IV CRC.

## Methods

### Research Objective

The primary objective of this study was to assess the survival advantage achieved by adding peri-operative chemotherapy to surgery in patients with resectable stage IV CRC.

### Searching for Trials

We performed electronic searches of Medline (PubMed), the Cochrane Library, and the Latin American and Caribbean Literature on Health Sciences (LILACS) between 1980 and 23 January 2009. We did not search Embase because we did not expect to retrieve any additional information [16].

We conducted the search using the following search terms based on medical subject headings (MeSH) and title words (TI): colorectal neoplasms (MeSH) and chemotherapy, adjuvant (MeSH) or anti-neoplastic combined chemotherapy protocols (MeSH) and neoplasm seeding (MeSH) or liver neoplasms/secondary (MeSH) or liver (TI) or hepatic (TI).

To avoid publication bias, both published and unpublished trials were identified through a computer-based search of the PubMed database and abstracts from the annual meetings of the American Society of Clinical Oncology. The set was limited to randomized clinical trials, clinical trials, and meta-analyses. No language restrictions were applied, thus reducing the potential for language bias. The search was also guided by a thorough examination of reference lists of original articles and review articles.

### Selection of Trials

We included only those trials in which patients were randomly assigned to at least two arms (surgery with peri-operative chemotherapy or surgery alone) and included only patients with pathologically-proven CRC who were to undergo curative resection.

Due to the small number of studies, one abstract was also included [17].

Trials initially designed to randomly assign patients to surgery, followed by different chemotherapy regimens in both arms, were considered ineligible [18,19].

### Assessment of study quality

The quality of all included studies was examined using the following criteria of the Cochrane Collaboration: generation of a random sequence; allocation concealment; blinding of patients, therapists, or outcome assessors; analysis of outcomes by intention-to-treat (ITT) and pre-specification of a primary endpoint. Each criterion was scored as follows: adequate, inadequate or unclear. Quality was assessed in duplicate by two independent reviewers.

### Data Abstraction

To avoid bias in the data abstraction process, two observers (SS and MW) independently abstracted the data from the trials and compared the results. The following information was culled from each report: year of publication; number of patients; gender; resection status; chemotherapy regimen; OS; RFS specific toxicity data; performance status and if available, treatment compliance.

In general, treatment compliance was defined as the number of patients who received the intended chemotherapy as a percentage of all the assessable patients.

The same two investigators repeated data extraction after some weeks in order to check for concordance. No relevant differences were noted.

Our meta-analysis was based solely on published data. Although we sought to obtain additional information from the principal investigators of the trials to confirm or update the published data, source data were unavailable.

### Quantitative Data Synthesis

The HRs were calculated to estimate how many times lower the probability of death from any cause was in patients receiving peri-operative chemotherapy after surgery compared with patients undergoing surgery alone. The HR provided in the report was used wherever available with 95% CIs [11,15,17,20].

In two studies, the crude log HR and its variance were calculated using the abstracted survival probabilities at each time-point from the Kaplan-Meier (KM) curves, using the IPD reconstruction technique [9,12].

We preferred the IPD reconstruction technique over the Parmar method [21] because it was easier to apply and similarly precise in small sample sizes, such as in the present trials [22]. Since the survival curves in these articles contained information on all events (i.e., downward steps) and censorings (i.e., spikes), this allowed us to extract data with precision. In addition, we recalculated p values and median survival times in order to determine whether or not the results correlated well with the original results.

In studies in which no survival curves were available, the relative risk (RR) was calculated from published incidence data. The RR denotes a measure of the risk of a certain event happening in one group compared to the risk of the same event occurring in another group, but without detailed consideration of follow-up times and completeness [10,13]. For statistical analyses, HR and RR were used synonymously. The natural logarithm of the HR (log HR) was used as effect size, with statistical weight inversely proportional to the variance of log HR. Since the HR was estimated to assess the survival advantage conferred by peri-operative chemotherapy a HR below unity was taken to indicate that adjuvant chemotherapy after surgery was superior to surgery alone.

Meta-analysis was first performed using fixed-effect modelling, which assumes that the treatment effect is theoretically the same in all studies. A subgroup meta-analysis was performed for systemic therapy and locoregional therapy. In the case of heterogeneity, however, random-effect modelling was selected, which assumes that each study result is randomly distributed around the true value. Heterogeneity testing was performed by calculating the Q and I^2 ^statistic. We considered heterogeneity to be present if the I^2 ^statistic was > 50%.

Potential sources of between-trial heterogeneity were explored by sensitivity analyses focusing on pre-specified explanatory variables, such as the type of therapy (locoregional vs. systemic) and trial quality.

In addition, we examined how strong the overall results were influenced by each single trial. This was done by consecutively omitting every study from the meta-analysis (leave-one-out procedure, Table 1).

**Table 1:** Leave-one-out procedure

	All studies included	Without Nordlinger	Without Lorenz	Witout Portier	Without Langer	Without Kemeny	Without Rudroff	Without Lygidakis	Without Wagman
OS (HR)	0.94	NA	0.88	0.98	0.95	0.89	0.93	1.02	0.94
RFS (HR)	0.77	0.75	0.71	0.78	0.76	0.78	0.76	0.80	0.77

Abbreviations: HR: Hazard ratio

OS: overall survival; RFS: recurrence-free survival

To minimise the effects of publication bias, we performed a thorough search for unpublished studies, and used Begg's funnel plot as an analytical tool to quantify the potential presence of publication bias.

**Figure 1:**
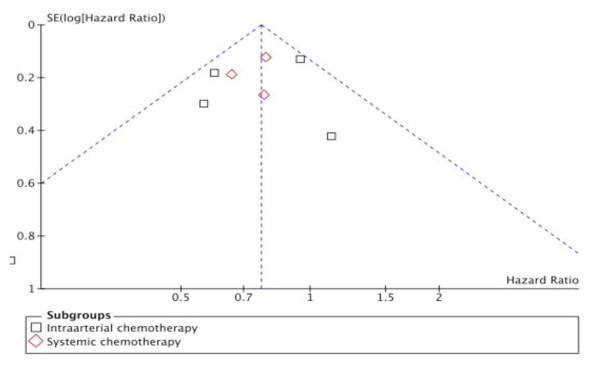
**Funnel-plot**. Funnel-plot with the Wagman trial included.

**Figure 2:**
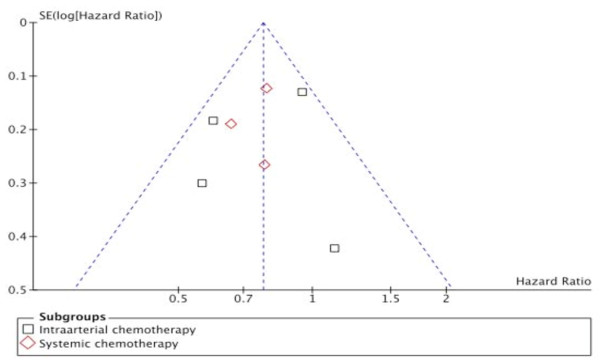
**Funnel - plot**. Funnel-plot without the Wagman trial.

When we plotted the overall estimates against the variance (sample size), a skewed asymmetric funnel was observed in the presence of the Wagman trial (Figure 1), which as a rather small study with small sample size (and large variance), would be more prone to publication bias, while a rather symmetric funnel was formed in the absence of the Wagman trial (Figure 2)

All analyses were performed using Review Manager (RevMan, version 5.0; Cochrane Collaboration, Oxford, England).

We defined a statistical result with a *p *value < .05 as significant.

## Results

### Patient characteristics

The available demographic characteristics of the 1174 patients entered into the trials are listed in Table 2.

**Table 2:** Patient characteristics

	Nordlinger	Lorenz	Portier	Langer	Kemeny	Rudroff	Lygidakis	Wagman
Number of pts recruited	364	226	173	129	109	42	40	91
Age (years)	Median 63	median 61	< 65: 102 ≥ 65: 69	< 70: 86 ≥ 70: 21	median: 60	median 58	mean: 61,5	mean: 57,8
Male/female	241/ 119 (+4)	126/100	99/72 (+2)	70/37 (+22)	67/40 (+2)	18/24	33/7	52/39
Number of metastases	1-4	1-6	1-4 (> 4 in 8 pts)	1-4	1-3	1-4 (> 4 in 3 pts)	n.r.	1
PS	WHO ≤ 2	Karnofsky 90 - 100%	WHO < 2	WHO ≤ 2	ECOG ≤ 2	n.r.	n.r.	Karnofsky > 50%

Abbreviations: n.r. not reported

PS: Performance status according to inclusion criteria

There were 706 males and 438 females. The gender of 30 patients was not further specified [9,15,17,20].

Randomized patients had a median/mean age of < 65 years. The number of metastases varied between one and six. In one study, only solitary metastases were included [13].

### Trial flow

Our main database search produced 271 potentially relevant references, of which 50 full articles were selected. Additionally, screening of reference lists led to two trials published in abstract form [17,23]. All the other searches yielded no additional, potentially relevant articles. The trial by Wagman was a six-armed trial. Arms A1 and A2 compared chemotherapy after surgery with surgery alone and comprised a total of 11 patients. These two arms were treated as an individual trial and included in our analysis [13].

Most of the potentially relevant articles had to be excluded because of having a non-randomised design [24] or comparing two different chemotherapeutic regimens [18,19]. We excluded the trial by Kemeny et al. [25] because survival data were not available. One of the remaining nine trials was only published in abstract form and did not contain sufficient information [23].

Thus, data from eight trials were included in this meta-analysis.

### Study characteristics

The baseline characteristics of the eight trials are listed in Table 3.

**Table 3:** Characteristics and quality of studies

	**Nordlinger**, 2008	Lorenz, 2005	Portier, 2006	Langer, 2002	Kemeny, 2002	**Rudroff**, 1998	Lygidakis, 1995	Wagman, 1990
Therapy regimen	FOLFOX 4, systemic	5-FU/FS, HAI	5-FU/FS, systemic	5-FU/FS, systemic	FUDR, HAI + 5-FU/FS, systemic	Mitomycin C + 5FU/FS, HAI	Immuno-therapy + 5-FU/FS, HAI	FUDR, HAI
Patients analyzed (n=)	364	226	171	107	109	30 **	40	11 *
Chemotherapy	182	113	86	52	53	14	20	5
Control	182	113	85	55	56	16	20	6
Received assigned treatment (n=)	250 (68,7%)	134 (59,3%)	136 (79,5%)	83 (77,6%)	65 (59,6%)	29 (96,6%)	39 (97,5%)	n.r.
Completed chemotherapy (n=)	neoadjuvant: 143 adjuvant: 80	34	54	28	20	13	19	n.r
Underwent surgery	170	100	82	55	45	16	20	6
Randomisation	adequate	adequate (tel. periop)	adequate	n.r.	adequate (preop)	adequate (postop)	adequate	adequate (preop)
R0 - resection	303	189	171	107	unclear	30	37	11
Concealed allocation	Unclear	unclear	unclear	n.r.	unclear	unclear	adequate	unclear
Blinded evaluation	Unclear	unclear	unclear	n.r.	unclear	unclear	unclear	unclear
Analysis on ITT basis	adequate	adequate	adequate	n.r.	inadequate	adequate	adequate	adequate
Primary endpoint	PFS	OS	DFS (2 yrs)	OS	RFS (4 yrs)	OS	OS	TTF
Secondary endpoint	Toxicity	Intrahepatic recurrence	OS	DFS	OS	RFS	Intrahepatic recurrence	OS; RR
Follow-up (Months)	45	18	87.4	-	51	144	-	-
Quality	High	high	high	low	low	low	low	low

* The total number of trial patients exceeds the sum of patients in groups due to multi-arm design.

** The total number of trial patients exceeds the sum of patients in groups because 12 patients with Dukes A/B were not randomized in this trial.

Abbreviations. pre: randomisation took place pre-operatively, post: randomisation took place

Post-operatively; Peri: randomisation took place during surgery;

Twenty - two patients from the Langer trial [17] and two patients from the Portier trial [20] were excluded from analysis because there were no complete post baseline data available. Twelve patients from the Rudroff trial [12] were not randomized and therefore excluded. Due to multi - arm design eighty patients from the Wagman trial were also excluded from our analysis [13]. In total, 1058 patients were analyzed, 525 patients who were randomized to surgery with peri-operative chemotherapy and 533 patients who were randomized to surgery alone. Five studies included the majority of patients (977 of 1058 [92.3%]) [9,11,15,17,20].

In the peri-operative chemotherapy arm, 5 FU/FA was applied systemically in two trials including 278 patients [17,20] and via continous hepatic artery infusion (cHAI) in one trial [11].

In other trials, 5FU/FA formed the basis for other chemotherapy regimens with different drugs added [9,10,12]. FOLFOX 4 was used in one large trial, including 364 patients [15]. One trial used cHAI of FUDR only [13]. Peri-operative chemotherapy was applied mainly as adjuvant treatment, either systemic or regional, or both. One trial was designed to administer pre- and post-operative chemotherapy [15].

Although in all studies the main eligibility criterion was resectable liver metastasis of colorectal origin, the definition of resectability varied among studies. Surgical procedures consisted of anatomic and non-anatomic resections in all studies. Since patients were randomised before surgery in most trials, metastasis were defined as potentially R0. Post-operative resection status was reported in seven trials, comprising a total of 848 patients. In the Kemeny trial [9], R0 resection status was not explicitly stated.

Patients in the trials by Portier et al. and Langer et al. [20,17] were evaluated monthly throughout the adjuvant treatment period.

Thereafter patient follow-up was performed every three months for two years, then once per year until death or the end of the study in the Portier trial [20]. In the Langer trial patients were evaluated three and six months after the end of treatment and then every six months until five years from randomization [17]. In the trial by Kemeny et al. [9] patients were observed during and after therapy every three months for the first three years. In the trial by Rudroff et al. [12] patients were examined at three months intervals during the first and second year after surgery, every six months during the subsequent three years and on a yearly basis thereafter.

In the trial by Lorenz et al. [11] follow-up examinations were performed every three months for the first two years after surgery and every six months thereafter. In the Nordlinger trial [15] follow-up examinations were performed after three cycles of chemotherapy, every three months for the first two years after the end of treatment and every six months thereafter.

In the trial by Lygidakis et al. [10] patient follow-up was performed every three months for the first year, at four months intervals during the second year and at six months intervals during the following years.

Wagman [13] did not provide any information about follow-up intervals.

We assessed the quality of all the trials using the criteria of the Cochrane Collaboration. There was a statement on both randomization and withdrawal in most of the trials. However, none of the trials were described as double-blind.

Trials treating at least one-half of patients with the assigned therapy, which reported an intent-to-treat analysis and accrued a large number of patients, were considered high quality [11,15,20].

### Overall survival

In all eight studies, OS was either a primary or secondary outcome variable. In the trial by Nordlinger et al. [15] OS has not yet been reported. The results of all the studies included indicated no reduction in overall mortality attributable to the administration of chemotherapy (HR, 0.94; 95% CI, 0.8-1.10; *p *= 0.43). This result was homogeneous (I^2 ^= 26%). Looking at the results in the pre-specified subgroups, trials involving intra-arterial chemotherapy failed to show a survival benefit (HR, 1.0; 95% CI, 0.84-1.21; *p *= 0.96; I^2 ^= 30%).

With respect to the four trials in which R0 resections were reported [10-13], the results of HAI chemotherapy did not significantly change (HR, 0.95; 95% CI, 0.78-1.16; p = 0.6).

The two trials on systemic chemotherapy failed to provide significant improvement in long-term OS compared with surgery alone in the initial analysis as single trials.

**Figure 3:**
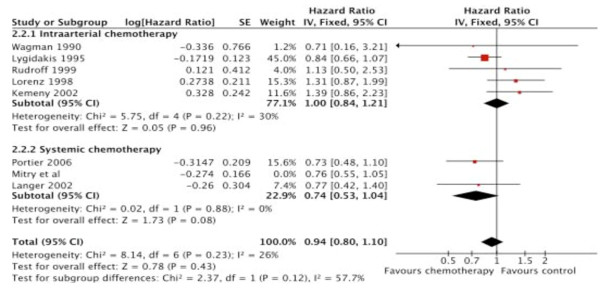
**Overall Survival**. Overall survival with peri-operative chemotherapy compared with surgery alone. The summary hazard ratio (HR) was 0.94 (95% CI, 0.80-1.10; *P *= 0.43) for peri-operative chemotherapy compared with surgery alone.

Our meta-analysis showed a firm tendency towards better OS for the systemic therapy subgroup (HR, 0.74; 95% CI, 0.53-1.04; *p *= 0.08; I^2 ^= 0%; Table 4, Figure 3).

**Table 4:** Overall Survival (OS)

	Nordlinger	Lorenz	Portier	Langer	Kemeny	Rudroff	Lygidakis	Wagman
Median OS time (months)								

Chemotherapy	-	34.5	62.1	53	34.2	-	20	37.3

Control	-	40.8	46.4	43	47.5	-	11	28.3

OS at last follow-up (cumulative %)								

Chemotherapy	-	-	51.1	57	37	25	95	-

Control	-	-	41.1	47	49.3	31	80	-

Hazard Ratio	-	1.31^3^	0.73^3^	0.77^3^	1.39 ^1^	1.13^1^	0.84^2^	0.71^2^

SE	-	0.21	0.21	0.30	0.24	0.41	0.12	0.77

P value	-	0.15	0.13	0.39	0.19	0.77	< 0.001	0.66

^1 ^HRs were calculated based on the IPD reconstruction technique

^2 ^only RR could be abstracted

^3 ^HRs were abstracted from original reports

Because both treatment modalities were combined in the Kemeny et al. trial [9], we included this trial in the systemic therapy subgroup for one separate calculation. The results showed a decreased chance of improvement with systemic therapy alone from 26% to only 8% (HR, 0.92; 95% CI, 0.7-1.21; p = 0.54), which might reflect the possible negative effects of HAI observed in other HAI trials.

### Recurrence-free survival

**Figure 4:**
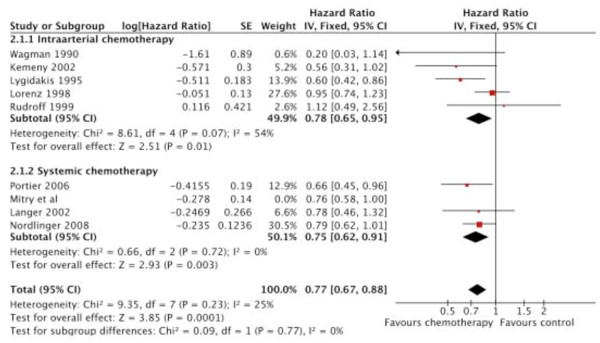
**Recurrence-free survival**. Recurrence-free survival with peri-operative adjuvant chemotherapy compared with surgery alone. The summary hazard ratio (HR) was 0.77 (95% CI; 0.67- 0.88; *P *= 0.0001) for peri-operative chemotherapy compared with surgery alone.

At first, the fixed effect model was used for meta-analysis. The results showed a significant improvement for the peri-operative chemotherapy group as a whole (HR, 0.77; 95% CI, 0.67-0.88; p = 0.0001), with the effect being similar in both subgroups (HR, 0.78; 95% CI, 0.65-0.95; *p *= 0.01 for HAI; and HR, 0.75; 95% CI, 0.62-0.91; *p *= 0.003 for systemic therapy; Table 5, Figure 4).

**Table 5:** Recurrence - free survival (RFS)

	Nordlinger	Lorenz	Portier	Langer	Kemeny	Rudroff	Lygidakis	Wagman
Median RFS time (months)								
Chemotherapy	18.7	21.6	24.4	39	-	-	-	30.7
Control	11.7	24.0	17.6	20	-	-	-	8.7
RFS at last follow-up (cumulative %)								
Chemotherapy	35.4	33.3	33.5	45	45.7	15	100	-
Control	28.1	36.7	26.7	35	25.2	23	55	-
Hazard Ratio/RR	0.79^3^	0.95^2^	0.66^3^	0.78^3^	0.56^1^	1.12^1^	0.6^2^	0.2^2^
SE	0.13	0.13	0.19	0.27	0.3	0.42	0.18	0.89
P value	0.058	0.72	0.028	0.35	0.04	0.79	< 0.001	0.03

^1 ^HRs were calculated based on the IPD reconstruction technique

^2 ^only RR could be abstracted

^3 ^HRs were abstracted from original reports

The heterogeneity was minimal between all of the included studies (I^2 ^= 25%).

Considering only the three trials of higher quality (HR, 0.82; 95% CI, 0.70-0.96) [11,15,20], the results did not differ significantly compared to all eight trials (HR, 0.77; 95% CI, 0.67-0.88)

Despite a suboptimal regime which was standard at the time recruitment started, the two randomized trials investigating the effect of systemic adjuvant therapy with 5 FU/FA showed a trend towards a better RFS in one study (39 months vs. 20 months; HR, 0.78; 95% CI, 0.46-1.32; p = 0.35) [17], and a significant improvement in RFS in the other trial (24.4 months vs. 17.6 months; HR, 0.66; 95% CI, 0.46-0.96; p = 0.028) [20]. The Nordlinger trial [15] tested pre-and post-operative chemotherapy with FOLFOX 4 versus surgery alone. The study failed to demonstrate a significant improvement in RFS for the peri-operative chemotherapy group in the intent-to-treat population, which might in part be due to the fact that in the EORTC trial [15], only 63% of patients received the intended post-operative treatment.

Subgroup analysis included all three trials and revealed a significant benefit in RFS (HR, 0.75; 95% CI, 0.62-0.91; p = 0.003) for the systemic therapy group.

In the trial by Kemeny at al. [9], systemic as well as locoregional treatment modalities were combined. RFS and recurrence in the liver were significantly reduced with manageable toxicities. The 25% risk reduction of recurrence attained with systemic therapy was similar when the Kemeny trial [9] was included in this subgroup (HR, 0.73; 95% CI, 0.6 1-0.88; p = 0.0007).

On the contrary, in the first interim analysis of the German trial on adjuvant HAI chemotherapy, Lorenz et al. [11] reported a median survival of 34.5 months for the adjuvant HAI therapy group compared with 40.8 months for the surgery group; the median time to progression was not improved. Because of possible harmful effects of HAI observed in this analysis, further accrual of patients was stopped prematurely. These discrepant results might be due to the fact that the German study used a different chemotherapy regimen (FUDR vs. FU/FS) and a different schedule (5 days vs. 28 days). It remains unclear how much systemic therapy really adds to the positive results reported by Kemeny et al. [9].

Subgroup analysis of all five trials using regional therapy showed a significant benefit in RFS conferred by HAI (HR, 0.78; 95% CI, 0.65-0.95; p = 0.01).

Because heterogeneity between studies in the HAI subgroup was high (I^2 ^= 54%), we applied the random effects model to estimate the overall treatment effect of locoregional treatment on RFS.

After adjustment for heterogeneity, results were no longer significant (HR, 0.72; 95% CI, 0.51-1.02; *P *= 0.07).

When only those trials which reported clear R0 resections were taken into account, the HR for RFS in the HAI subgroup did not fundamentally change (HR, 0.81; 95% CI, 0.67-1.00) compared with all five trials in this subgroup.

### Toxicity

The most frequent grade 3 and 4 toxicities of peri-operative chemotherapy are listed in Table 6.

**Table 6:** Most frequent grade 3 to 4 toxicities of peri-operative chemotherapy

	Nordlinger	Lorenz	Portier	Langer	Kemeny	Rudroff	Lygidakis	Wagman
Assessable patients (n =)	115*	73	81	n.r.	30	n.r.	20	11
Gastrointestinal toxicity								+
Diarrhea	6	10	7	n.r.	1	n.r.	-	
Stomatitis	-	35	6	n.r.	-	n.r.	1	
Nausea/Vomiting	8	25	6	n.r.	5	n.r.	2	
Hepatic	6	7	-	n.r.	8	n.r.	-	
Hematologic toxicity			6					+
Leucopenia	14	-	-	n.r.	2	n.r.	-	
Neutropenia	40	-	-	n.r.	-	n.r.	3	
Thrombopenia	8	-	-	n.r.	1	n.r.	-	
Neurologic toxicity								+
Sensory neuropathy	11	-	2	n.r.	-	n.r.	-	
Fever							19	
Treatment-related death	0.	8	0	n.r	0	n.r.	0	2

Abbreviations:

* only the toxicity of post-operative chemotherapy is reported here

+ in this trial only general information about toxicity is provided

n.r. not reported

The toxicity profiles were obtained for 330 patients (62.9%) of the 525 patients assigned to the chemotherapy arms.

Toxicities were generally mild and acceptable. Grade 3-4 leucopenia and grade 3-4 neutropenia were observed in 4.9% and 13% of patients, respectively. Grade 3 or more severe nausea and vomiting, diarrhea, and hepatic toxicity were observed in 13.9%, 7.3%, and 6.4% of the patients who received peri-operative chemotherapy.

Langer and Rudroff [12,17] did not provide any information about possible side effects of chemotherapy, while Wagman [13] reported grade 4 gastrointestinal, hematologic, and neurologic toxicity without further specification.

In the Nordlnger trial [15], peri-operative complications occurred more often in the chemotherapy group (25% vs. 16%, p = 0.04). Mortality rates were low in both groups and there was no suspected chemotherapy-associated deaths in this trial.

The pump placement showed no effect on operative complications in the Kemeny trial [9], whereas Lorenz et al. [11] reported a 7.5% early (within 30 days following surgery) treatment-related mortality rate for the HAI chemotherapy group.

Altogether, there were 10 treatment-related deaths (12%) in two trials on intrahepatic chemotherapy [11,13]

## Discussion

In the present meta-analysis, we have included, for the first time, several randomized controlled trials (RCTs) involving stage IV CRC in which a patient group with peri-operative treatment was compared with a group undergoing surgery alone.

The peri-operative chemotherapy was delivered via different routes of application (systemically, intra-arterially or both).

Considering all included trials, the HR estimates suggested that peri-operative chemotherapy yielded no survival advantage over surgery alone (HR, 0.94; 95% CI, 0.8-1.10; *p *= 0.43). With respect to RFS, the peri-operative treatment group had a significant survival benefit (HR, 0.77; 95% CI, 0.67-0.88; *p *= 0.0001).

We undertook further subset analyses due to the different routes of application.

With respect to systemic therapy, the clinical use of adjuvant chemotherapy in stage III colorectal cancer is well-established, so one may well think that patients with resected stage IV CRC might also benefit from adjuvant treatment. However, in this cancer stage a formal proof of a better OS was still missing. Existing trials had to close prematurely because of slow accrual. A control arm for surgery alone was needed, but difficult to obtain [26].

As a result of our subset analysis, we were able to show that systemic chemotherapy yielded a significant survival benefit concerning RFS for the patient group with peri-operative systemic therapy compared to surgery alone in patients with resected stage IV CRC.

The results showed a clear tendency towards better OS after systemic adjuvant therapy with bolus 5FU/FA. The OS data from the Nordlinger trial [15] are expected in 2010.

As subset analysis has thus demonstrated the usefulness of systemic chemotherapy, the question remains whether or not locoregional chemotherapy would be as beneficial for operable patients as systemic chemotherapy.

Since the liver is the most common site of recurrence after resection of colorectal liver metastases, locoregional adjuvant treatment might be a reasonable treatment modality, but its widespread use is limited for several reasons. The application of regional chemotherapy to the liver requires arterial catheter placement, which is frequently associated with catheter complications, such as infections, hematomas, or thromboses, meaning in-hospital stays for several days or even weeks and substantial discomfort for the patient. Moreover, locoregional chemotherapy does not control occult systemic metastasis.

According to our analysis, the results at first showed a significant improvement in RFS for the locoregional chemotherapy group (HR, 0.78; 95% CI, 0.65-0.95; *P *= 0.01).

After adjustment for heterogeneity, the results were no longer significant (HR, 0.72; 95% CI, 0.51-1.02; *P *= 0.07). OS was not improved.

Our results are thus in agreement with other analyses. In 2006, Nelson [27] considered seven randomized trials addressing this issue with locoregional treatment. He found no survival benefit for the group that received HAI. Even though two trials applied additional systemic chemotherapy, the results were slightly in favour of the control group (8.9% survival advantage), although this effect was not significant (HR, 1.089; 95% CI, 0.887-1.334). In the pooled analysis by Mitry et al. [14], two studies were considered. The investigators found a marginal statistical benefit in favor of adjuvant therapy concerning median progression free survival (p = 0.058). In evaluating OS, however, they could only demonstrate a non-significant improvement (p = 0.095).

Yet several problems remain unresolved.

Peri-operative chemotherapy can be further divided into two clearly defined treatment modalities (the pre-operative [neoadjuvant] treatment and the post-operative [adjuvant] treatment). Since the EORTC trial [15] was intended to demonstrate that chemotherapy combined with surgery is a better treatment than surgery alone, and not to compare pre- vs. post-operative chemotherapy, the exact role of pre-operative chemotherapy in the case of initially resectable liver metastases remains unknown and cannot be answered with data from the aforementioned trials.

Additionally we analyzed patients with partially unknown post-operative resection status (R0 vs. R1) who received different types of chemotherapy regimens as one group.

The heterogeneity among trials in the HAI subgroup may be a limitation of our meta-analysis, even though we applied a random-effect model that takes possible heterogeneity into consideration.

Although the study by Nordlinger et al. [15] included more patients than the previously reported pooled analysis by Mitry et al. [14] and employed a more effective regimen, we found no evidence of study heterogeneity in relation to the graphical or statistical methods within the systemic therapy group.

Regarding the role of FOLFIRI as another effective chemotherapy regimen in the adjuvant treatment of CRC, the study by Ychou et al. [18] showed no significant improvement in disease-free survival (DFS) compared with 5FU/FA.

Grade 3/4 toxic effects were more common in patients treated with FOLFIRI vs. 5 FU/FA (47% vs. 30%), with neutropenia being the most common (23% vs. 7%).

Thus, further clarification of which patient group would benefit by peri-operative chemotherapy, whether applied pre- or post-operatively, and which drug or combination of drugs would be most effectively applied, is essential.

The results of further studies will hopefully elucidate the most suitable treatment modality in operable patients.

Although compliance to systemic treatment appeared to be acceptable in the trials that were included in our analysis (66.7% and 63% of the planned treatments) [15,20], further efforts to improve chemotherapeutic regimens to minimize toxicities are clearly warranted.

Some technical aspects should be mentioned in relation to this meta-analysis.

All our analyses were based on abstracted data and not on individual patient data (IPD). The results must therefore be interpreted cautiously, as an IPD-based meta-analysis would give a more reliable estimation than one based on abstracted data.

Additionally, for reasons of general applicability, we chose RFS as the common endpoint of our analysis.

Assuming a R0 resection in all patients, we considered it appropriate to translate the respective endpoints of the analyzed trials, such as progression-free survival (PFS) or disease-free survival (DFS) into recurrence - free survival (RFS).

In the original report, DFS was calculated from the time of resection until recurrence or death of any cause [20]. Langer [17] did not provide an exact definition of DFS.

Kemeny [9] chose the 4-year recurrence-free rate as a primary endpoint in her trial and calculated from the date of randomisation. In the trial by Rudroff et al. [12] the recurrence-rate was a secondary outcome variable estimated from the date of liver resection. The secondary outcome variable recurrence in the liver was calculated from the date of randomisation in the Lorenz trial [11]. Lygidakis [10] reported on intrahepatic recurrence without further time to event assessment. PFS was counted from the date of randomisation until progressive disease or recurrence or death of any cause in the Nordlinger trial [15].

Follow-up intervals were similar in all except one trial.

The respective endpoint in the six-armed Wagman trial [13] was TTF and was calculated from the day of surgery. Here, TTF was chosen because the other groups (arms B1, B2, C1, und C2) were not rendered disease-free at the time of randomisation. In this trial no further information about follow-up intervals was provided.

Yet in spite of some differences in the respective endpoints, between-study variability was quite low among all the eight trials which were included (I^2 ^= 25%).

The inclusion of results presented in the abstract form, which may be only preliminary, might also have biased our final results. However, since our analysis of Langer and Portier [17,20] correlated well with the pooled analysis by Mitry et al. [14], which was based on IPD, any bias due to this factor is likely to be small.

The accuracy of the HRs estimated from the survival curves is another important issue. We obtained fairly good correlation between the HRs reported in the articles and those obtained based on the published survival curves, suggesting that curve-based HRs can be substituted in cases where the HRs are not available.

## Conclusions

In conclusion, this is the first meta-analysis to demonstrate the benefit of systemic peri-operative chemotherapy in the treatment of patients with resected stage IV CRC. Even though the strength of our main conclusion was limited by the fact that it was based on abstracted data, peri-operative chemotherapy should be recommended in case of initially resectable metastases of CRC.

Our results should be confirmed by an IPD-based meta-analysis.

## Competing interests

D. Arnold: Honoraria: Roche, Sanofi, Pfizer, Amgen, Merck. Research funding: Roche, Sanofi, Pfizer.

W. Schmiegel: Honoraria: Merck, Roche, Abbott, Amgen, Pfizer, Falk. Research Funding: Roche, Sanofi-Aventis. Travel Support: Roche, Merck, Astra-Zeneca. Advisory role: Roche, Amgen, Astra-Zeneca.

A. Reinacher-Schick: Honoraria: Amgen, Roche, Sanofi, Pfizer. Research funding: Roche, Sanofi.

## Authors' contributions

MW participated in the design and coordination of the study, performed the statistical analysis and drafted the manuscript. ARS conceived of the study, participated in its design and helped to draft the manuscript. SS performed the statistical analysis. DA participated in the design of the study and helped to draft the manuscript WS conceived of the study and participated in its design. All authors read and approved the final manuscript.

## Pre-publication history

The pre-publication history for this paper can be accessed here:

http://www.biomedcentral.com/1471-2407/10/309/prepub
